# Tartary Buckwheat (*Fagopyrum tataricum*) NAC Transcription Factors FtNAC16 Negatively Regulates of Pod Cracking and Salinity Tolerant in *Arabidopsis*

**DOI:** 10.3390/ijms22063197

**Published:** 2021-03-21

**Authors:** Jing Wang, ZhaoTang Ma, Bo Tang, HaoYu Yu, ZiZhong Tang, TongLiang Bu, Qi Wu, Hui Chen

**Affiliations:** College of Life Science, Sichuan Agricultural University, Ya’an 625000, China; JingWang507@163.com (J.W.); WaohUncle_Ma@163.com (Z.M.); tb1638857231@163.com (B.T.); yhy2413203899@163.com (H.Y.); 14126@sicau.edu.cn (Z.T.); tlbu@163.com (T.B.); wuqi@sicau.edu.cn (Q.W.)

**Keywords:** pod cracking, salt-stress, lignin, FtNAC16

## Abstract

The thick and hard fruit shell of *Fagopyrum tataricum* (*F. tataricum*) represents a processing bottleneck. At the same time, soil salinization is one of the main problems faced by modern agricultural production. Bioinformatic analysis indicated that the *F. tataricum* transcription factor FtNAC16 could regulate the hull cracking of *F. tataricum*, and the function of this transcription factor was verified by genetic transformation of *Arabidopsis thaliana* (*A. thaliana*). Phenotypic observations of the wild-type (WT), OE-FtNAC16, *nst1/3* and *nst1/3*-FtNAC16 plant lines confirmed that FtNAC16 negatively regulated pod cracking by downregulating lignin synthesis. Under salt stress, several physiological indicators (POD, GSH, Pro and MDA) were measured, *A. thaliana* leaves were stained with NBT (Nitroblue Tetrazolium) and DAB (3,3’-diaminobenzidine), and all genes encoding enzymes in the lignin synthesis pathway were analyzed. These experiments confirmed that FtNAC16 increased plant sensitivity by reducing the lignin content or changing the proportions of the lignin monomer. The results of this study may help to elucidate the possible association between changes in lignin monomer synthesis and salt stress and may also contribute to fully understanding the effects of FtNAC16 on plant growth and development, particularly regarding fruit pod cracking and environmental adaptability. In future studies, it may be useful to obtain suitable cracking varieties and salt-tolerant crops through molecular breeding.

## 1. Introduction

Pod cracking is a common way for plants to reproduce, ensure the prosperity of the race and enhance plant adaptability [1,2]. However, premature cracking and complete noncracking of fruit pods and shells may affect the yield of crops in agricultural production, such as rape, soybean and castor [1,3,4,5]. On the other hand, to handle increasingly severe environmental changes (e.g., high temperature and soil salinization), crops need to regulate their own development [6]. Therefore, it is necessary to cultivate good crop varieties with high stress resistance and high yields by ensuring proper cracking of fruit pods and considering the growth conditions of plants under stress.

Plants enter an adaptation stage after they are stimulated by stress signals, at which point they activate the defense stress system, and after a short time the plant metabolism undergoes profound remodeling [7,8]. These changes are undertaken to enable plants to withstand damage from stressful environments and long-term exposure to stress, while maintaining important biological processes [6]. Regarding plant responses to salt stress, the role of transcription factors in stress signals and defense stress systems has been studied deeply. Although there are some studies on metabolic remodeling, this topic still needs to be further clarified [9,10].

High salt stress causes changes in plant primary and secondary metabolism [11]. Recent studies have observed that transcription factors play positive roles in abiotic stress tolerance by regulating lignin synthesis in plants, such as SiMYB56 and AgNAC1 [12,13,14]. In addition, a recent study has determined that the expression levels of most of the genes encoding key enzymes in the lignin pathways of salt-adapted cells are increased compared with normal cells, and the study also demonstrated that *ccoaomt1-1* and *ccoaomt1-2* were phenotypically hypersensitive to salt stress [15]. Although the accumulation of lignin and the strong expression of lignin biosynthesis genes in plants during the maintenance stage are key to salt adaptation, few studies have investigated the gene expression pattern changes in lignin monomer pathway enzymes under salt stress.

NAC proteins involved in secondary cell wall (SCW) biosynthesis include three *NST* genes (*NST1*, *NST2* and *NST3/SND1*) [16]. Previous studies of these genes have focused on SCW synthesis and pod shattering [5,17,18]. Lignin is the main component of the SCW, controlled by NAC-MYB regulatory networks [19]. *NST1-3* is considered the main switch, and *NST1* and *NST3* have functional redundancy [20]. In summary, the main switching factors of SCW synthesis may play important roles in coordinating development and the stress response by regulating lignin metabolism.

*F. tataricum* is an economically important crop that can be utilized in medicine and food, and through the publication of its genome, promoting this plant as a model plant for the study of resistance [21]. Some key abiotic stress response transcription factors of *F. tataricum* have been identified [22,23], but no lignin pathway has been involved in these studies. Based on the main switch, FtNAC16, in the lignin synthesis pathway, our research identified this protein as the key factor regulating the husking of *F. tataricum* and analyzed the relationship between lignin metabolism regulation and salt tolerance. The additional roles played by transcription factors related to *F. tataricum* shelling in environmental adaptation were demonstrated from a new perspective, providing important theoretical support for genetic engineering to enhance the shelling/pod cracking and stress resistance of crops in the future.

## 2. Results

### 2.1. Isolation and Analysis of FtNAC16

**Figure 1:**
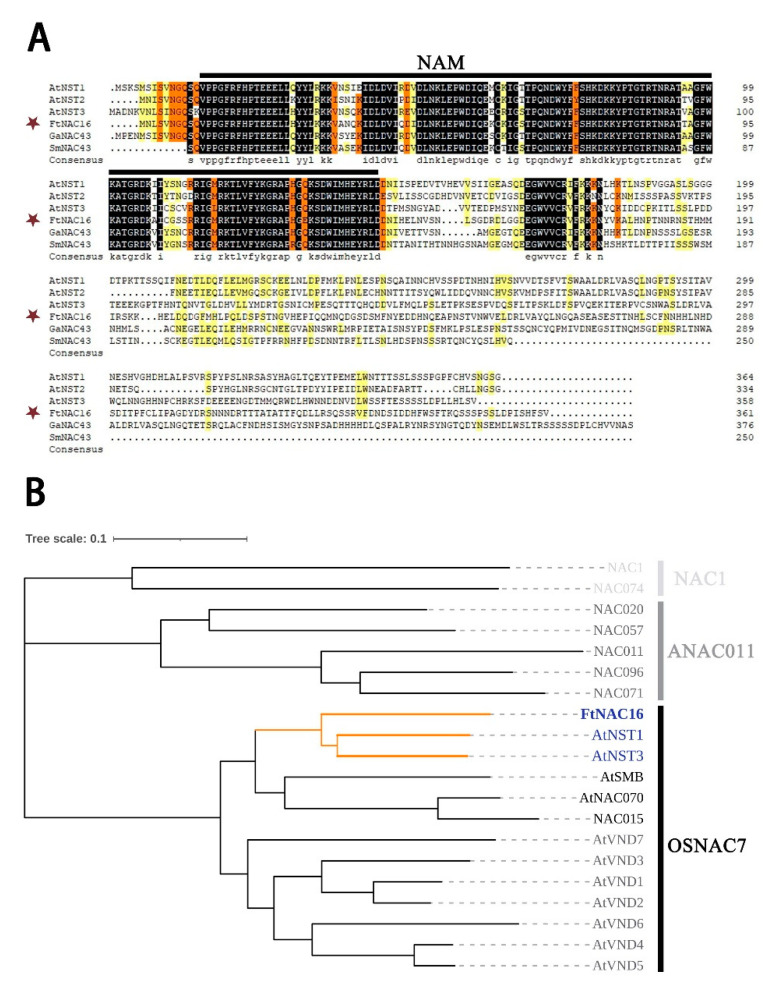
Sequence characteristics and homology analysis of FtNAC16. (**A**) Sequence alignment of the FtNAC16 protein and its conserved domain, represented by the black line. The GenBank accession numbers of the NAC protein areas were as follows: AtNST1 (ANAC043) (AT2G46770), AtNST2 (AT3G61910), AtNST3 (AT1G32770), FtNAC16 (FtPinG0000381200.01), GaNAC43 (XP_017629605.1) and SmNAC43 (QBZ39066.1). (**B**) Evolutionary tree analysis divides 20 NAC family genes into three subfamilies: NAC1, ANAC011 and OSNAC7. FtNAC16 and AtNST1/3 are clustered into the same branch and are shown in orange.

The *FtNAC16* gene is located on chromosome 1 of *F. tataricum*. Furthermore, the *FtNAC16* full-length genomic sequence includes one exon, and the open reading frame (ORF) of this gene is 1086 bp. The FtNAC16 protein contains 361 amino acids (AAs) (Figure 1A), including a typical NAM domain (Figure 1A). The theoretical isoelectric point (pI) of FtNAC16 is 6.04, and its molecular weight (MW) is 41.38 kD. Phylogenetic analysis show that *FtNAC16* is homologous to *AtNST1* and *AtNST3* (Figure 1B). *AtNST1* and *AtNST3* have functional redundancy in secondary wall synthesis [20]. We surmise that *FtNAC16* and *AtNST1/3* may perform similar functions.

### 2.2. Effects of FtNAC16 on Pod Cracking in A. thaliana

**Figure 2:**
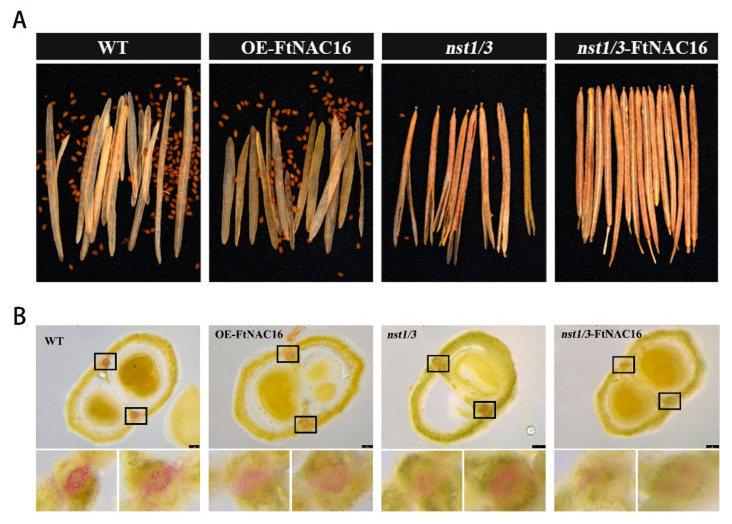
Dehiscence of four genotypes of *A. thaliana* and lignin deposition patterns in the cracking area. (**A**) Phenotypic map of mature fruit pods of wild-type (WT), OE-FtNAC16, *nst1/3* and *nst1/3*-FtNAC16; (**B**) *A. thaliana* fruit pods were cross-cut, lignin was stained with phloroglucinol, and the cracking area of the black box was locally enlarged.

To verify the function of FtNAC16 transcription factors in plants, we constructed transgenic *A. thaliana* lines. Different cracking states in the same strain were not found when the fruit pod matured; Figure 2A represents the cracking state of the whole fruit pod. Phenotypic observations demonstrated that there was no significant difference between OE-FtNAC16 and WT plants with regards to fruit pod cracking (Figure 2A and Figure S1), neither in the time of cracking nor the degree of cracking. Considering the functional redundancy of *nst1* and *nst3* in *A. thaliana*, we obtained *nst1/3* double mutants from the TAIR library (https://www.arabidopsis.org/ (aaccessed on 15 March 2021)). When we transferred *FtNAC16* into the mutant *nst1/3,* the pods did not crack at all (Figure 2A). This result indicated that *FtNAC16* may play a negative regulatory role in fruit pod cracking.

To verify whether the cracking of *A. thaliana* was due to a difference in lignin synthesis, we made transverse sections and stained them with phloroglucinol (Figure 2B). The results showed that there was no significant difference between OE-FtNAC16 and WT regarding lignin accumulation in the replum of fruit, but the lignin accumulation in *nst1/3* and *nst1/3*-FtNAC16 was clearly lower, especially in the latter, which contained almost no lignin. Thus, *NST1/3* was demonstrated to play a key role in regulating pod cracking, whereas *FtNAC16* appears to negatively regulate pod cracking in *A. thaliana* by reducing lignin synthesis.

### 2.3. Expression Patterns of FtNAC16 under Salt Stress Treatments

**Figure 3:**
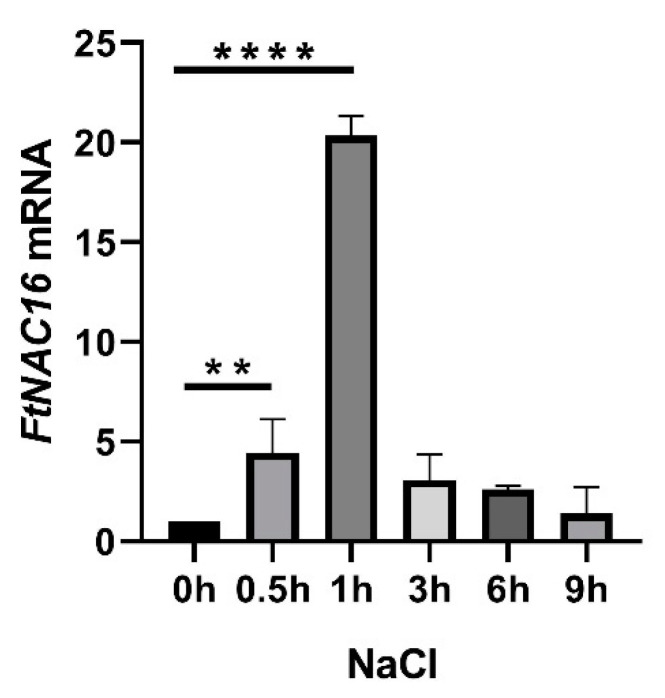
Relative expression of the *FtNAC16* transcript in *F. tataricum* subjected to salt stress. The relative expression of the *FtNAC16* transcript in *F. tataricum* was determined by means of qRT-PCR. Two-week-old seedlings were used to extract mRNA following treatment with 200 mM NaCl for 9 h. The sampling times were 0, 0.5, 1, 3, 6 and 9 h. The error bars indicate the standard error (SE) of three replicates. ** *p* <0.01, **** *p* <0.0001.

We analyzed *FtNAC16* promoter elements and found that they contain ABRE elements and TC-rich repeats (defense stress elements). The expression patterns of *FtNAC16* after *F. tataricum* salt stress were analyzed by means of qPCR. The peak appeared at 1 h and later began to decrease after 200 mM NaCl stress treatment (Figure 3. *FtNAC16* may be involved in the response of *F. tataricum* to salt stress. We also treated *F. tataricum* with ABA. The results showed that the expression of *FtNAC16* began to decrease 1 h after ABA treatment, and the expression level was the lowest after 3 h (Figure S2). These results indicate that ABA may inhibit *FtNAC16* expression.

### 2.4. FtNAC16 Promotes Root Elongation under Salt Stress

**Figure 4:**
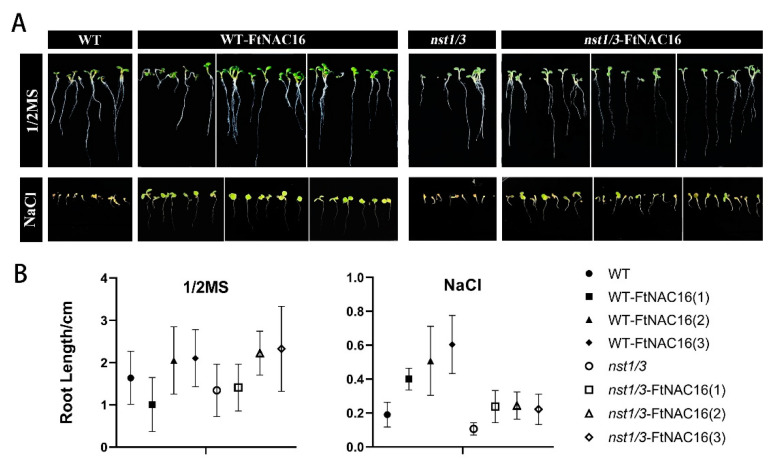
The influence of *FtNAC16* to root elongation under salt stress. Phenotypic (**A**) and length analysis (**B**) of the effects of 1/2 MS and salt stress on the root elongation of WT, OE-FtNAC16, *nst1/3* and *nst1/3*-FtNAC16 lines. The error bars represent ± SDs, and each data value is from three replicate experiments.

Under salt stress, plant roots first sensed stress signals and produced corresponding physiological responses, which subsequently affected the growth of the aboveground plant parts. Root length experiments showed that there was no significant difference between plants on 1/2 MS medium (Figure 4A). In the saline environment, root development was inhibited in all plant lines; among the lines, *nst1/3* exhibited the strongest root development. OE-FtNAC16 and *nst1/3*-FtNAC16 were observed to promote root elongation relatively (Figure 4B).

### 2.5. Overexpression of FtNAC16 Increased Salt Sensitivity in A. thaliana

**Figure 5:**
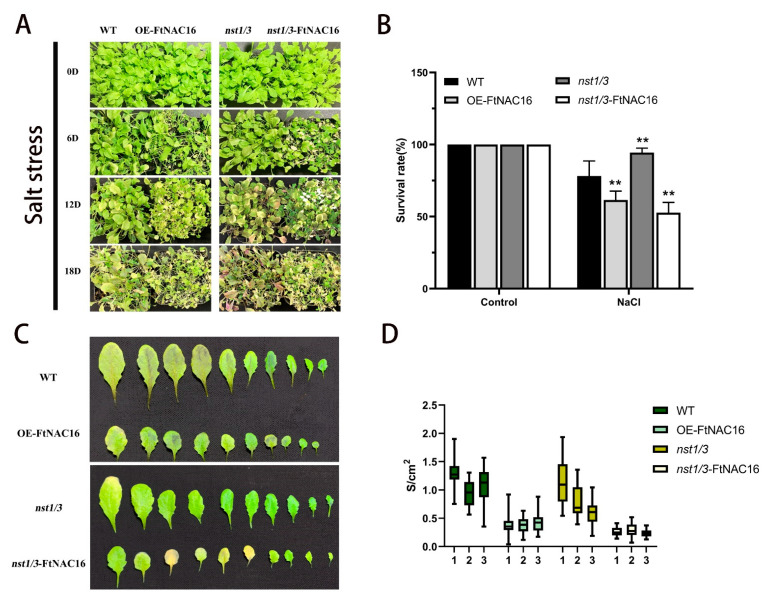
Phenotypic status of the four genotypes after salt stress treatment. (**A**) Growth status of *A. thaliana* at 0, 6, 12 and 18 days under salt stress; (**B**) After salt stress treatment for 18 days, the survival rates of transgenic plants were counted containing three biological replicates; ** represents *t*-test significance: *p* < 0.01.; (**C**) effects of stress on leaf growth of *A. thaliana* in four genotypes after 18 days; (**D**) by measuring leaf length and width, blade size was calculated based on S = πab, 25 random repeat samples. The error bars indicate the standard error (SE) of three replicates.

To explore the mechanism governing the expression of this gene in response to salt stress during the vegetative growth period of *A. thaliana*, we treated *A. thaliana* under the same growth conditions (one-month-old seedlings) with salt stress. Phenotypic changes were observed after 6, 12 and 18 days of treatment with 150 mM NaCl salt stress (Figure 5A). Figure 5B shows that the survival rate of overexpression lines decreased notably. We also treated the *nst1/3* mutant and *nst1/3*-FtNAC16 in the same way and found that the survival rate of the *nst1/3* mutant was significantly higher than that of the WT. After the addition of *FtNAC16*, the survival rate of the plants decreased sharply, indicating that *FtNAC16* overexpression is not conducive to plant tolerance to high-salt environments. Equally importantly, we observed significant phenotypic differences in leaf development from stress phenotypes (Figure 5C); therefore, we statistically analyzed leaf size on the 12th day of salt stress treatment (Figure 5D).

The results showed that the leaf development of the OE-FtNAC16 and *nst1/3*-FtNAC16 lines was severely inhibited by the overexpression of *FtNAC16*. In summary, the salt stress tolerance of *nst1/3* was the highest, but the salt tolerance decreased significantly with *FtNAC16* overexpression. Accordingly, we conclude that *FtNAC16* increases salt sensitivity in *A. thaliana*.

### 2.6. Effects of FtNAC16 on the Physiological Status of Plants under Salt Stress

**Figure 6:**
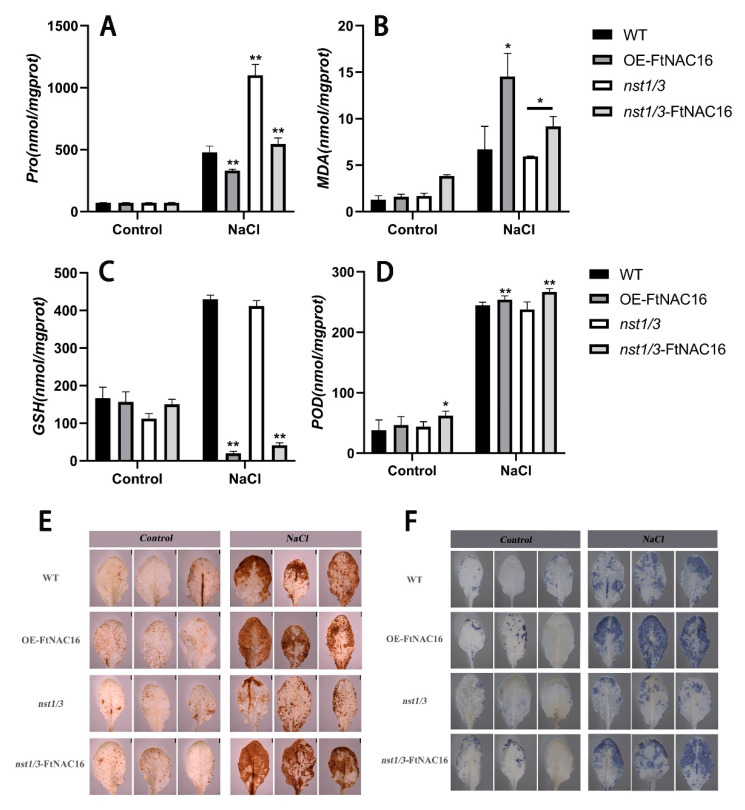
Effects of FtNAC16 on the physiological status of plants under salt stress. (**A**) The content of proline; (**B**) The content of MDA; (**C**) The content of GSH; (**D**) The content of POD; (**E**) The accumulation of H_2_O_2_ in the leaves of WT and transgenic lines was analyzed by means of histochemical staining with DAB under normal conditions and abiotic stress conditions, respectively. (**F**) The accumulation of O_2^−^_ in the leaves of WT and transgenic lines was analyzed by means of histochemical staining with NBT under normal conditions and abiotic stress conditions, respectively. The error bars denote ± SDs, and each data value is from three replicate experiments. At least 10 plants were used per experiment per line. * represents *t*-test significance: *p* < 0.05; ** represents *t*-test significance: *p* < 0.01.

Proline contents were measured for each transgenic plant line, as well as for WT plants, after 12 days of salt treatment. The proline content of the overexpression lines was significantly lower than that in WT plants. However, the proline content in the mutants was the highest, and when we recharged *FtNAC16*, the proline content decreased (Figure 6A). Malondialdehyde (MDA) was utilized as a measure of membrane damage, and the results showed that membrane damage was more severe when *FtNAC16* was overexpressed (Figure 6B). NBT (Nitroblue Tetrazolium) staining results showed that the content of superoxide anion in the overexpression strain increased. In contrast, the content of superoxide anion in the *nst1/3* mutant was the lowest, indicating that the superoxide anion in the mutant was well removed and protected from toxicity (Figure 6E). The results of POD content measurement (Figure 6D) and DAB (3,3’-diaminobenzidine) staining (Figure 6F) showed that the content of peroxidase in the leaves increased slightly after *FtNAC16* overexpression, but GSH, as a peptide in the non-enzymatic reaction, decreased sharply with *FtNAC16* overexpression. These results may help to explain the decrease in high-salt tolerance in plants overexpressing *FtNAC16* (Figure 6C). Overall, according to plant physiological indicators (MDA, Pro, POD, NBT and DAB staining), *FtNAC16* reduces stress tolerance in plants, although not by reducing POD enzyme activity.

### 2.7. Effects of FtNAC16 on Lignin Synthesis under Salt Stress

**Figure 7:**
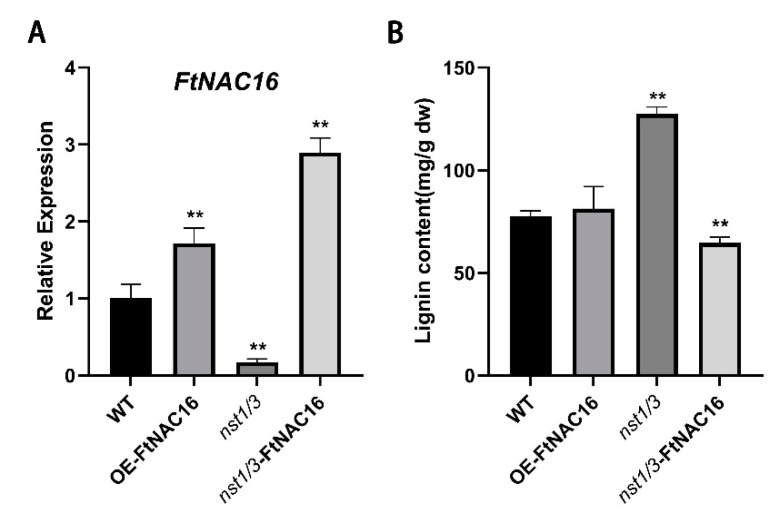
Effects of *FtNAC16* expression on lignin synthesis under salt stress. (**A**) Relative expression of the *FtNAC16* transcript in *A. thaliana* subjected to salt stress. (**B**) The content of lignin in the leaves of WT, OE-FtNAC16, *nst1/3* and *nst1/3*-FtNAC16 lines after salt stress. Significant differences are denoted by **, meaning *p* < 0.01.

Due to the key role played by *FtNAC16* in the lignin synthesis pathway and the newly identified relationship between lignin synthesis and salt tolerance, we have to consider the effect of *FtNAC16* on lignin synthesis under salt stress. We determined the *FtNAC16* expression and lignin content of four genotypes of *A. thaliana* under salt stress by qPCR and chemical methods. Thus, under salt stress, the lignin content of OE-FtNAC16 was not significantly different in the lines (Figure 7A). The lignin content of *nst1/3* was almost 1.5 times that of WT, and the lignin content in *nst1/3*-FtNAC16 decreased significantly (Figure 7B). These results demonstrate a negative regulatory relationship between *FtNAC16* expression and lignin synthesis under salt stress. We also observed that the expression of *FtNAC16* in *nst1/3*-FtNAC16 was higher than that in OE-FtNAC16 (Figure 7A). Higher expression levels of this gene lead to lower survival (Figure 5B) and more severe leaf growth inhibition (Figure 5D), again demonstrating the negative regulatory effect of *FtNAC16* on salt stress tolerance.

### 2.8. Changes in the Lignin Pathway after Salt Stress

**Figure 8:**
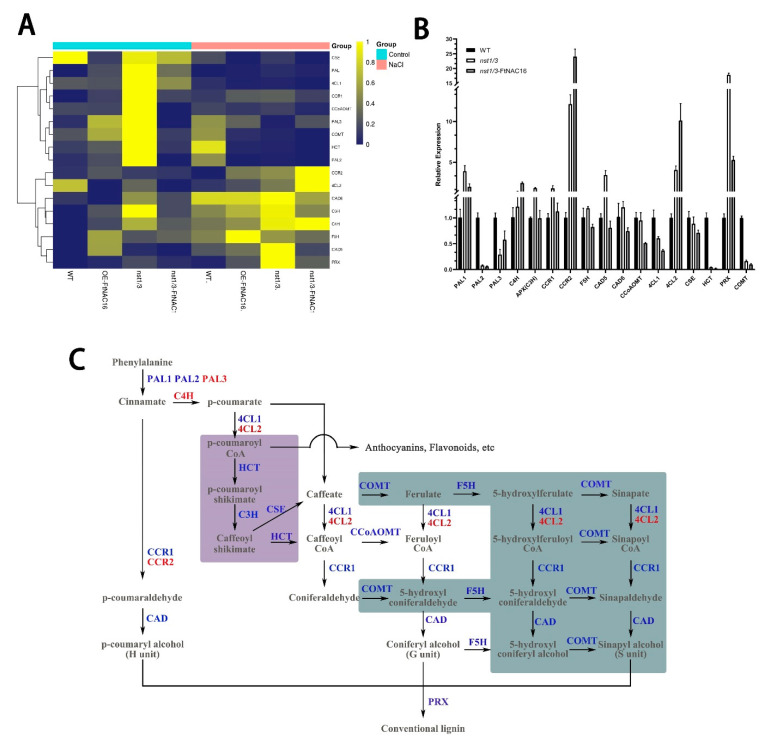
Expression of key enzyme genes in the lignin pathway after salt stress treatment. (**A**) Heat map of lignin pathway enzyme gene expression after salt stress treatment. Blue indicates upregulation, and yellow indicates downregulation. (**B**) Expression of lignin synthase genes in WT, *nst1/3* and *nst1/3*-FtNAC16 lines after salt stress; (**C**) Lignin synthesis pathway pattern. The blue text represents downregulated enzyme genes in *nst1/3*-FtNAC16, and the red font represents upregulated genes compared to *nst1/3* lines. The purple background and green background depict inhibited branches in *nst1/3* and *nst1/3*-FtNAC16 lines under salt stress (compared with WT).

To determine whether the adaptability of *nst1/3* mutants to salt stress and *FtNAC16*-induced a salt sensitivity change due to changes in lignin pathway gene expression, qPCR methods were used to analyze the expression of key enzyme genes in the lignin pathway (Figure 8A). First, after salt stress treatment, most of the lignin pathway enzyme genes showed upregulated expression in the WT plants; these genes included *PAL1, CCR1, PAL3, COMT, HCT, PAL2, CAD6, APX, C4H, F5H* and *CAD5*. In the OE-FtNAC16, *nst1/3* and *nst1/3*-FtNAC16 lines, enzyme genes were clearly divided into two categories. The nine genes in the first branch mostly exhibited downregulation after salt treatment, and the eight genes in the second branch were upregulated. Second, we found that *COMT*, *HCT* and *PAL2* were upregulated in WT but downregulated in the other three genotypes, indicating that the pathway by which WT plants and the other three genotype plants synthesize lignin under salt stress was changed, and this difference was caused by *NST1/3* genes.

Under salt stress, *FtNAC16* appears to perform the opposite function as *AtNST1/3* in the lignin pathway, and the function of *FtNAC16* in the OE-FtNAC16 line is weakened because of *AtNST1/3*. The total lignin detection results also confirmed this result. Next, we compared WT, *nst1/3* and *nst1/3*-FtNAC16 plants and examined the changes in the lignin pathway in the three lines under the salt environment (Figure 8B). *HCT*, *COMT* and *PAL2* were downregulated in *nst1/3* and *nst1/3*-FtNAC16. In *nst1/3*, there were no differences in the expression of *CCoAOMT* compared with WT, but *CCoAOMT* expression was downregulated in *nst1/3*-FtNAC16. COMT and F5H are essential enzymes for the synthesis of S-lignin monomer; these were down-regulated in *nst1/3*-FtNAC16 (Figure 3). *COMT* was also downregulated in overexpression lines (Figure S3). However, the expression of *C4H*, *CCR2* and *4CL2* was upregulated (Figure 8B and Figure S3). These results show that the synthesis pathway of lignin tends to synthesize H units and G units (Figure 8C). Thus, *FtNAC16* was determined to inhibit S unit synthesis under salt stress.

We found that *HCT* was involved in a branch of the lignin synthesis pathway (purple background). However, the expression of *HCT* in *nst1/3* and *nst1/3*-FtNAC16 was almost non-existent (Figure 8B, C). The expression of *HCT* in the OE-FtNAC16 line was also strongly downregulated under salt stress (Figure S3). These results indicated that under salt stress, lignin synthesis was mediated not through p-coumarate–p-coumaroyl shikimate–caffeate but directly through p-coumarate–caffeate. All lignin pathway enzyme genes, except *C4H*, *PAL3*, *CCR2* and *4CL2*, were downregulated after overexpressing *FtNAC16* in the *nst1/3* plant line; of these genes, the abnormally upregulated expression of *4CL2* and *CCR2* may represent functional compensation provided by redundant genes.

The highest expression levels of *CAD5*, *CAD6* and *PRX* were observed in *nst1/3*, and significantly downregulated expression was observed in *nst1/3*-FtNAC16. CAD is the last key enzyme to synthesize the lignin monomer. PRX is a key enzyme in the polymerization of the lignin monomer into macromolecules, suggesting that *nst1/3* plants synthesize more lignin under salt stress. The *nst1/3*-FtNAC16 synthesis of lignin decreased, which is consistent with the results obtained through lignin content measurement (Figure 7B). Overall, *FtNAC16* was observed to negatively regulate the expression of the lignin synthase gene, thereby inhibiting lignin synthesis under salt stress.

The results of this study indicate that *FtNAC16* negatively regulates lignin synthesis, affects *A. thaliana* pod cracking resistance, and alters the lignin synthesis pathway during metabolic remodeling under salt stress. Furthermore, *FtNAC16* was observed to inhibit S unit synthesis and reduce the total amount of lignin synthesis.

## 3. Discussion

Moderate cracking of fruit pods in many crops is very important and can bring many benefits. Lignin deposition in the pod cleavage region is a key factor affecting its cracking [1,18]. Previous studies have shown that *nst1/3* plants exhibit a cracking-prone phenotype in *A. thaliana* (microcracking, easy shedding of seeds after twisting with fingers) [20,24], which is consistent with our observed *nst1/3* phenotype. OE-FtNAC16 was not a significant differential phenotype compared with WT in pod cracking. Furthermore, *FtNAC16* over-expression accentuated the phenotype of *nst1/3* mutants. There are two redundant genes in WT. *FtNAC16* does not weaken the phenotype caused by *AtNST1/3*, and after the deletion of these two genes, the phenotype of *FtNAC16* is highlighted, suggesting that *FtNAC16* performs functions that are contrary to *NST1/3* of *A. thaliana* in terms of pod cracking. Through comparative transcriptomics and WGCNA (Weighted Gene Co-Expression Network Analysis), the key module genes that make *F. tataricum* plants difficult to dehull have been located in the secondary wall synthesis network [25]. In this module, *FtNAC16* (an *NST1/3* homologous gene in *A. thaliana*) acts as one of the key upstream switching factors. Our experiments show that *FtNAC16* plays a negative regulatory role in fruit pod cracking, which may establish a theoretical foundation for future research investigating potential transcription factors that may facilitate the dehulling of *F. tataricum* and the fruit pod cracking of related crops.

Our results confirmed the positive role played by total lignin content in the salt stress response. At the same time, the change in the lignin pathway leads to a change in the monomer ratio, which may also be an important factor affecting salt stress. Lignin metabolic pathways are closely related to other metabolic processes, and changes in the expression of single or multiple lignin synthesis genes affects the phenotype of transgenic plants [26]. The lignin content of *A. thaliana* decreased, whereas monomer composition changes and plant growth were severely inhibited, in *cad*, *ccr* and *c4h* mutants [27,28]. Changing the biological pathway of lignin may often lead to plant dwarfing or abnormal development [29,30]. OE-FtNAC16 and *nst1/3*-FTNAC16 plants exhibit the phenotypes of very small leaf areas and stunted dwarf symptoms after salt stress treatment, which may be associated with FtNAC16-induced changes in lignin pathways. Although *nst1/3* has undergone changes in the lignin pathway, it exhibits notable salt tolerance due to its advantages in total lignin synthesis. This total lignin may contain more G units than S units, as the lignin pathway flows more to the G unit synthesis pathway. In summary, *FtNAC16* overexpression not only reduced the total content of lignin and affected the salt tolerance of plants but also affected the lignin synthesis pathway under salt stress.

Syringyl lignin (S unit) biosynthesis requires F5H and COMT involvement [31]. Inhibiting the expression of *F5H*, *COMT* and *CCoAOMT* causes a change in the lignin S/G ratio, which contributes to G monomer synthesis, and the total lignin content also decreases [32,33]. The ratio of S units to S/G in the HCT-RNAi (RNA interference of *HCT* gene) line of poplars was observed to decrease, whereas the G units increased [34]. A recent study reported that the total amount of lignin and the S unit in the cell wall of maize increased significantly after salt stress [35]. This finding is consistent with the salt sensitivity of overexpression lines but is inconsistent with the synthesis of G units in *nst1/3* plants. Therefore, we believe that the total amount of lignin is the primary factor, and the role of the lignin monomer is second. Our experimental results show that changing the synthesis pathway of the lignin monomer effects plant salt sensitivity, which provides a basis for the role played by the lignin monomer ratio in adapting to salt stress. Of course, determining whether the proportion of the lignin monomer is random or relevant for salt tolerance warrants further study.

Some studies have shown that the inhibition of root length is related to the increase in lignin content [36,37]. FtNAC16 promoted root elongation under salt stress, which may be related to the negative regulation of lignin content mediated by *FtNAC16*. Moreover, in OE-FtNAC16, *nst1/3* and *nst1/3*-FtNAC16 plants, among the inhibited *HCT* branching pathways, *C3H* and *CSE* have been reported in recent studies to be involved in the reactive oxygen species (ROS) mechanism in addition to lignin synthesis [38]. In this branch, *CSE* and *C3H* and *CSE* were also downregulated in *nst1/3*. The mutant plants were downregulated further after rescue. *C3H* was most highly expressed in mutants after overexpressing *FtNAC16*. This branch seems to be unable to participate in the synthesis of lignin under salt stress in OE-FtNAC16, *nst1/3* and *nst1/3*-FtNAC16. *CSE* and *C3H* may be involved in functions other than the lignin pathway. Therefore, *NST1/3* may play roles in coordinating both pathways under salt stress.

Consistent with the results of previous studies [39], the expression of *ABI4* was significantly increased after salt treatment, and the highest expression level was found in *nst1/3* plants. Meanwhile, the expression of *FtNAC16* in *F. tataricum* plants decreased after ABA signals were received, indicating that excessive ABA signaling may inhibit *FtNAC16* expression (Figure S4). Hence, we surmise that FtNAC16–ABI4–NCED3–ABA–FtNAC16 may exhibit a positive feedback mechanism in synthetic regulatory networks. This positive feedback mechanism exacerbates the function of *FtNAC16* in high-salt environments, leading to metabolic remodeling in plants that cannot maintain normal growth. Indeed, previous studies reported that positive feedback regulation did exist between ABA and *NCED3* under drought stress and confirmed that this mechanism was not mediated by *ABF3* [40]. Our hypothesis complements the ABA positive feedback loop under osmotic stress and provides a possible research direction for studies attempting to elucidate the mechanisms governing *NST1/3* in response to osmotic stress.

**Figure 9:**
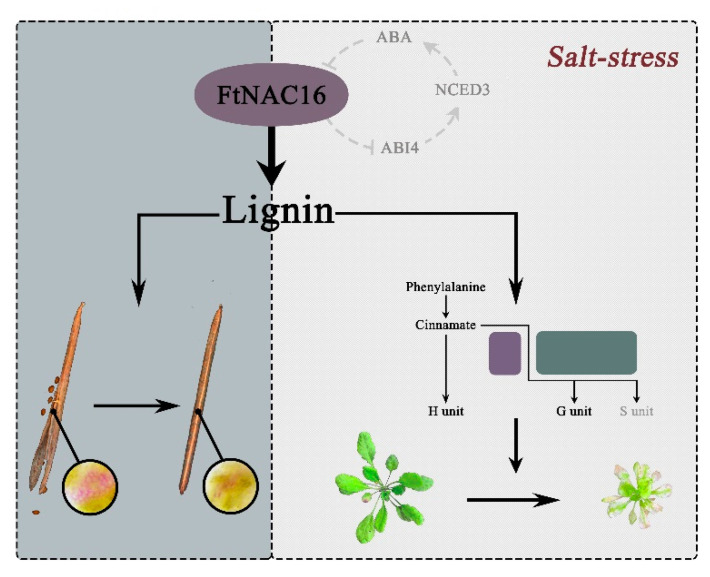
Functional model of FtNAC16. FtNAC16 not only regulates pod cracking through the lignin pathway, but also affects salt stress tolerance. The gray background represents the effect of FtNAC16 on the cracking of *A. thaliana* plant pods through the lignin pathway, the beige background shows the effect of FtNAC16 on the tolerance of salt stress, and the line ring depicts the hypothesis regarding FtNAC16 and ABA pathways. Short lines indicate inhibition; dashed lines indicate possible regulation.

According to the results of this study, a pattern diagram was built (Figure 9). In this model, FtNAC16 negatively regulates fruit pod cracking in normal development and plant tolerance under salt stress by regulating the lignin pathway, which may help to establish a theoretical foundation and molecular basis for hull cracking and salt tolerance in *F. tataricum* and other related crops.

## 4. Materials and Methods

### 4.1. Cloning of FtNAC16 cDNA from F. tataricum

Total RNA was extracted from the mixed tissues of *F. tataricum* and the synthesized first-strand cDNA was reverse-transcribed. Specific primers were designed to amplify the full-length open reading frame (ORF) of the *FtNAC16* gene by PCR.

(Forward primer 5′-ATGAATCTCTCAGTCAATGG-3′, Reverse primer 5′-ATACCGAGAAATGAGAGATTG-3′).

### 4.2. Bioinformatic Analysis

FtNAC16 homologous protein sequences were retrieved from the NCBI database according to our previous research on the family identification of NAC transcription factors in *F. tataricum* [41]. The multiple amino acid sequences obtained were calibrated and aligned using DNAMAN v6.0.3.99 (Lynnon Biosoft, SanRamon, CA, USA). Sequence information is provided in Table S1. Next, the neighbor-joining method was used to build a phylogenetic tree via the Mega 7.0 (Mega Limited, Auckland, New Zealand) program, and the conserved domains were predicted via the MEME website. Sequence information is provided in Table S2. The ExPASy bioinformatic resource portal was used to analyze the physical and chemical properties of FtNAC16.

### 4.3. Plant Materials

The seeds of *F. tataricum* “Rice-Tartary No. 1” were germinated on wet filter paper under long-day illumination (16 h light/8 h dark) at 23 °C. The germinated seeds were subsequently cultivated in 1/2-strength Hoagland solution for two weeks, after which they were treated with 150 μM ABA and 200 mM NaCl. Samples were collected after 0, 0.5, 1, 3, 6 and 9 h of treatment and stored at −80 °C until RNA was extracted. Untreated seedlings exhibiting similar growth and development were used as controls. Each sample consisted of three biological repeats.

Col-0 (WT) and *nst1/3* mutants were obtained from TAIR, and OE-FtNAC16 and *nst1/3*-FtNAC16 were obtained through the transformation of *A. thaliana* using floral dip method [42].

### 4.4. Determination of Lignin Content and Phloroglucinol-HCl Stain

UV spectrophotometry was used to determine lignin content. Phloroglucinol-HCl Stain: Two grams of resorcinol was dissolved in 80 mL of 20% ethanol solution, and 20 mL of 12 N (12 mol/L) HCl was added thereafter. Fresh tissue samples were cut into thin slices of 100–200-µM thickness. Next, samples were soaked in Phloroglucinol-HCl solution for 10–15 min, removed, placed on glass slides with clear water, covered with glass slides and observed and photographed under a microscope (Leica M205 FA, Leica Microsystems Ltd., Wetzlar, Hesse-Darmstadt, Germany).

### 4.5. Stress Tolerance Assays of Transgenic A. thaliana

In salt stress experiments, under identical growth conditions, T_3_ homozygous transgenic lines, *nst1/3* and WT plants were cultured in soil for three weeks. Three-week-old seedlings were treated with 200 mM NaCl for 18 days, and phenotypic changes were recorded every six days. Survival was calculated after 18 days of NaCl treatment. After 12 days of salt stress, POD and GSH activities of the four lines were determined. Simultaneous measurements of MDA and Pro after 12 days of salt treatment above physiological indicators were obtained using previously described methods [43]. In addition, DAB and NBT staining methods were used to evaluate ROS accumulation in leaves [22]. The above experimental treatments were repeated three times.

The root length experiment was carried out on 1/2 MS medium and 150 mM NaCl 1/2 MS. *A. thaliana* seeds were sterilized with 75% ethanol and 0.1% HgCl_2_. After 7 days of cultivation, the root length was measured. Three transgenic lines were biological repeats.

### 4.6. qPCR Analysis

Total RNA from samples was extracted and the synthesized first-strand cDNA (complementary DNA) was reverse-transcribed. Quantitative real-time PCR (qPCR) was performed using a TB Green Premix Ex Taq II Kit with a CFX96 RT-PCR machine (Bio-Rad, Hercules, State of California, United States of America). The amplification programs utilized in this analysis were as follows: 98 °C for 45 s, followed by 34 cycles of 98 °C for 15 s and 60 °C for 45 s. Each sample was analyzed in triplicate to ensure the accuracy of the data. *Atactin2* was employed as a housekeeping gene, and the primers involved in qPCR analysis are listed in Table S3.

### 4.7. Data Analysis

Microsoft Excel and Prism were employed for data processing and graphics rendering.

## 5. Conclusions

Our study has shown that FTNAC16 is an important transcription factor, which can negatively regulate *A. thaliana* pod dehiscence and increase plant sensitivity to salt. These phenotypes are attributable to changes in lignin synthesis pathways. The expression of *FtNAC16* and the homologous gene *NST1/3* in *A. thaliana* alters the lignin monomer synthesis pathway under salt stress. Furthermore, we observed that *A. thaliana nst1/3* mutants have higher salt stress tolerance than the wild type. To the best of our knowledge, this study is the first to describe these results. Finally, we also observed that there may be a positive feedback mechanism between ABA and FtNAC16 under salt stress. Our results not only help to establish a theoretical foundation for *F. tataricum* shell traits and environmental tolerance, but also provide future directions for research on other industrial crops which also have cracking problems. Further study is also warranted in order to characterize the relationship between the ABA pathway and FtNAC16.

## Data Availability

No new data were created or analyzed in this study. Data sharing is not applicable to this article.

## Supplementary Materials

The following are available online at https://www.mdpi.com/1422-0067/22/6/3197/s1, Figure S1. Phloroglucinol-HCl staining of *A. thaliana* fruit pods. Figure S2. Relative expression of the FtNAC16 transcript in *F. tataricum* subjected to ABA. Figure S3. Expression of key enzyme genes in the lignin pathway after salt stress treatment in WT and OE-FtNAC16. Figure S4. Expression of the *AtABI4* gene in the lignin pathway after salt stress treatment in WT, *nst1/3* and *nst1/3*-FtNAC16. ** *p* <0.01. Table S1. Alignment sequence. Table S2. Data of evolution tree. Table S3. q-PCR Primers.

## References

[1] Dong Y., Yang X., Liu J., Wang B.H., Liu B.L., Wang Y.Z. (2014). Pod shattering resistance associated with domestication is mediated by a *NAC* gene in soybean. Nat. Commun..

[2] Parker T., Berny Mier y Teran J.C., Palkovic A., Jernstedt J., Gepts P. (2019). Genetic control of pod dehiscence in domesticated common bean: Associations with range expansion and local aridity conditions. bioRxiv.

[3] Fan W., Lu J., Pan C., Tan M., Lin Q., Liu W., Li D., Wang L., Hu L., Wang L. (2019). Sequencing of Chinese castor lines reveals genetic signatures of selection and yield-associated loci. Nat. Commun..

[4] Liu J., Zhou R., Wang W., Wang H., Qiu Y., Raman R., Mei D., Raman H., Hu Q. (2020). A copia-like retrotransposon insertion in the upstream region of the *SHATTERPROOF1* gene, BnSHP1.A9, is associated with quantitative variation in pod shattering resistance in oilseed rape. J. Exp. Bot..

[5] Krisnawati A., Adie M.M. (2017). Identification of Soybean Genotypes for Pod Shattering Resistance Associated with Agronomical and Morphological Characters. Biosaintifika J. Biol. Biol. Educ..

[6] Steiner F. (2020). Plant Abiotic Stress Tolerance.

[7] Bhardwaj R., Handa N., Sharma R., Kaur H., Kohli S., Kumar V., Kaur P. (2014). Lignins and Abiotic Stress: An Overview. Physiological Mechanisms and Adaptation Strategies in Plants Under Changing Environment.

[8] Cabane M., Afif D., Hawkins S. (2012). Lignins and Abiotic Stresses. Adv. Bot. Res..

[9] Zhang X., Long Y., Huang J., Xia J. (2020). *OsNAC45* is Involved in ABA Response and Salt Tolerance in Rice. Rice (N. Y.).

[10] Gill S.S., Tuteja N. (2010). Reactive oxygen species and antioxidant machinery in abiotic stress tolerance in crop plants. Plant Physiol. Biochem..

[11] Cui G., Zhang Y., Zhang W., Lang D., Zhang X., Li Z., Zhang X. (2019). Response of Carbon and Nitrogen Metabolism and Secondary Metabolites to Drought Stress and Salt Stress in Plants. J. Plant Biol..

[12] Xu W., Tang W., Wang C., Ge L., Sun J., Qi X., He Z., Zhou Y., Chen J., Xu Z. (2020). *SiMYB56* Confers Drought Stress Tolerance in Transgenic Rice by Regulating Lignin Biosynthesis and ABA Signaling Pathway. Front. Plant Sci..

[13] Duan A.Q., Tao J.P., Jia L.L., Tan G.F., Liu J.X., Li T., Chen L.Z., Su X.J., Feng K., Xu Z.S. (2020). AgNAC1, a celery transcription factor, related to regulation on lignin biosynthesis and salt tolerance. Genomics.

[14] Li T., Huang Y., Khadr A., Wang Y.-H., Xu Z.-S., Xiong A.-S. (2020). DcDREB1A, a DREB-binding transcription factor from Daucus carota, enhances drought tolerance in transgenic *Arabidopsis thaliana* and modulates lignin levels by regulating lignin-biosynthesis-related genes. Environ. Exp. Bot..

[15] Chun H.J., Baek D., Cho H.M., Lee S.H., Jin B.J., Yun D.J., Hong Y.S., Kim M.C. (2019). Lignin biosynthesis genes play critical roles in the adaptation of Arabidopsis plants to high-salt stress. Plant Signal. Behav..

[16] Pascual M.B., de la Torre F., Cañas R.A., Cánovas F.M., Ávila C. (2018). NAC Transcription Factors in Woody Plants. Prog. Bot..

[17] Liu X., Tu B., Zhang Q., Herbert S.J. (2019). Physiological and molecular aspects of pod shattering resistance in crops. Czech J. Genet. Plant Breed..

[18] Zhang Q., Tu B., Liu C., Liu X. (2018). Pod anatomy, morphology and dehiscing forces in pod dehiscence of soybean (*Glycine max* (L.) Merrill). Flora.

[19] Zhao Q., Dixon R.A. (2011). Transcriptional networks for lignin biosynthesis: More complex than we thought?. Trends Plant Sci..

[20] Mitsuda N., Iwase A., Yamamoto H., Yoshida M., Seki M., Shinozaki K., Ohme-Takagi M. (2007). NAC transcription factors, NST1 and NST3, are key regulators of the formation of secondary walls in woody tissues of Arabidopsis. Plant Cell.

[21] Zhang L., Li X., Ma B., Gao Q., Du H., Han Y., Li Y., Cao Y., Qi M., Zhu Y. (2017). The Tartary Buckwheat Genome Provides Insights into Rutin Biosynthesis and Abiotic Stress Tolerance. Mol. Plant.

[22] Li Q., Wu Q., Wang A., Lv B., Dong Q., Yao Y., Wu Q., Zhao H., Li C., Chen H. (2019). Tartary buckwheat transcription factor FtbZIP83 improves the drought/salt tolerance of Arabidopsis via an ABA-mediated pathway. Plant Physiol. Biochem..

[23] Lv B., Wu Q., Wang A., Li Q., Dong Q., Yang J., Zhao H., Wang X., Chen H., Li C. (2020). A WRKY transcription factor, FtWRKY46, from Tartary buckwheat improves salt tolerance in transgenic *Arabidopsis thaliana*. Plant Physiol. Biochem..

[24] Zhong R., Ye Z.H. (2015). The Arabidopsis NAC transcription factor NST2 functions together with SND1 and NST1 to regulate secondary wall biosynthesis in fibers of inflorescence stems. Plant Signal. Behav..

[25] Li H.Y., Wu C.X., Lv Q.Y., Shi T.X., Chen Q.J., Chen Q.F. (2020). Comparative cellular, physiological and transcriptome analyses reveal the potential easy dehulling mechanism of rice-tartary buckwheat (*Fagopyrum Tararicum*). BMC Plant Biol..

[26] Yoon J., Choi H., An G. (2015). Roles of lignin biosynthesis and regulatory genes in plant development. J. Integr. Plant Biol..

[27] Schilmiller A.L., Stout J., Weng J.K., Humphreys J., Ruegger M.O., Chapple C. (2009). Mutations in the cinnamate 4-hydroxylase gene impact metabolism, growth and development in Arabidopsis. Plant J..

[28] Thevenin J., Pollet B., Letarnec B., Saulnier L., Gissot L., Maia-Grondard A., Lapierre C., Jouanin L. (2011). The simultaneous repression of *CCR* and *CAD*, two enzymes of the lignin biosynthetic pathway, results in sterility and dwarfism in *Arabidopsis thaliana*. Mol. Plant.

[29] Gui J., Shen J., Li L. (2011). Functional characterization of evolutionarily divergent 4-coumarate:coenzyme a ligases in rice. Plant Physiol..

[30] Shadle G., Chen F., Srinivasa Reddy M.S., Jackson L., Nakashima J., Dixon R.A. (2007). Down-regulation of hydroxycinnamoyl CoA: Shikimate hydroxycinnamoyl transferase in transgenic alfalfa affects lignification, development and forage quality. Phytochemistry.

[31] Wu Z., Wang N., Hisano H., Cao Y., Wu F., Liu W., Bao Y., Wang Z.Y., Fu C. (2019). Simultaneous regulation of *F5H* in *COMT*-RNAi transgenic switchgrass alters effects of *COMT* suppression on syringyl lignin biosynthesis. Plant Biotechnol. J..

[32] Li X., Weng J.K., Chapple C. (2008). Improvement of biomass through lignin modification. Plant J..

[33] Li X., Chen W., Zhao Y., Xiang Y., Jiang H., Zhu S., Cheng B. (2013). Downregulation of caffeoyl-CoA O-methyltransferase (CCoAOMT) by RNA interference leads to reduced lignin production in maize straw. Genet. Mol. Biol..

[34] Zhou X., Yang S., Lu M., Zhao S., Cai L., Zhang Y., Zhao R., Lv J. (2020). Structure and Monomer Ratio of Lignin in *C3H* and *HCT* RNAi Transgenic Poplar Saplings. ChemistrySelect.

[35] Oliveira D.M., Mota T.R., Salatta F.V., Sinzker R.C., Koncitikova R., Kopecny D., Simister R., Silva M., Goeminne G., Morreel K. (2020). Cell wall remodeling under salt stress: Insights into changes in polysaccharides, feruloylation, lignification, and phenolic metabolism in maize. Plant Cell Environ..

[36] Geng D., Chen P., Shen X., Zhang Y., Li X., Jiang L., Xie Y., Niu C., Zhang J., Huang X. (2018). MdMYB88 and MdMYB124 Enhance Drought Tolerance by Modulating Root Vessels and Cell Walls in Apple. Plant Physiol..

[37] Sharma N.K., Gupta S.K., Dwivedi V., Chattopadhyay D. (2020). Lignin deposition in chickpea root xylem under drought. Plant Signal. Behav..

[38] Dixon R.A., Barros J. (2019). Lignin biosynthesis: Old roads revisited and new roads explored. Open Biol..

[39] Jeong C.Y., Lee W.J., Truong H.A., Trinh C.S., Jin J.Y., Kim S., Hwang K.Y., Kang C.S., Moon J.K., Hong S.W. (2018). Dual role of SND1 facilitates efficient communication between abiotic stress signalling and normal growth in Arabidopsis. Sci. Rep..

[40] Yang Y.Z., Tan B.C. (2014). A distal ABA responsive element in *AtNCED3* promoter is required for positive feedback regulation of ABA biosynthesis in Arabidopsis. PLoS ONE.

[41] Liu M., Ma Z., Sun W., Huang L., Wu Q., Tang Z., Bu T., Li C., Chen H. (2019). Genome-wide analysis of the NAC transcription factor family in Tartary buckwheat (*Fagopyrum tataricum*). BMC Genom..

[42] Bent S.J.C.a.A.F. (1998). Floral dip: A simplified method for Agrobacterium-mediated transformation of *Arabidopsis thaliana*. Plant J..

[43] Kotchoni S.O., Kuhns C., Ditzer A., Kirch H.H., Bartels D. (2006). Over-expression of different aldehyde dehydrogenase genes in *Arabidopsis thaliana* confers tolerance to abiotic stress and protects plants against lipid peroxidation and oxidative stress. Plant Cell Environ..

