# Supplementary material for: Tartary Buckwheat (Fagopyrum tataricum) NAC Transcription Factors FtNAC16 Negatively Regulates of Pod Cracking and Salinity Tolerant in Arabidopsis
Source: Int J Mol Sci. 2021 Mar 21;22(6):3197. doi: 10.3390/ijms22063197 (PMC8061773; doi:10.3390/ijms22063197)
Supplement: Supplementary file 1 [file ijms-22-03197-s001.zip › Supplementary Table S1. Alignment sequence.pdf]

Supplementary Table S1. Homologous protein alignment sequence

Name	Gene ID	Sequence
AtNST1 (ANAC043)	AT2G46770	MNLSVNGQSQVPPGFRFHPTEEELLHYYLKKKVANQKI DLDVIQDIDLNKLEPWDIQEKCKIGSTPQNDWYFFSHKD KKYPTGTRTNRATTAGFWKATGRDKAICGSSRRIGMRK TLVFYKGRAPHGEKSDWIMHEYRLDDNIHELNVSNLSG DRDLGGDEGWVVCRVFRKKNYVKALHNPTNNRNSTH MMIRSKKHELDQDGFMHLPQLDSPSTNGVHEPIQQMNQ DGSDSMFNYEDDHNQEAPNSTVNWVELDRLVAYQLNG QASEASESTTNHLSCFNNHHLNHDSDITPFCLIPAGDYDR SNNNDRTTTATATTFQDLLRSQSSRVFDNDSIDDHFWSF TKQSSSPSSLDPISHFSV
AtNST2	AT3G61910	MNISVNGQSQVPPGFRFHPTEEELLKYYLRKKISNIKIDL DVIPDIDLNKLEPWDIQEMCKIGTTPQNDWYFYSHKDK KYPTGTRTNRATTVGFWKATGRDKTIYTNGDRIGMRKT LVFYKGRAPHGQKSDWIMHEYRLDESVLISSCGDHDVN VETCDVIGSDEGWVVCRVFKKNNLCKNMISSSPASSVK TPSFNEETIEQLLEVMGQSCKGEIVLDPFLKLPNLECHNN TTITSYQWLIDDQVNNCHVSKVMDPSFITSWAALDRLV ASQLNGPNSYSIPAVNETSQSPYHGLNRSGCNTGLTPDY YIPEIDLWNEADFARTTCHLLNGSG
AtNST3	AT1G32770	MADNKVNLSINGQSKVPPGFRFHPTEEELLHYYLRKKV NSQKIDLDVIREVDLNKLEPWDIQEECRIGSTPQNDWYF FSHKDKKYPTGTRTNRATVAGFWKATGRDKIICSCVRRI GLRKTLVFYKGRAPHGQKSDWIMHEYRLDDTPMSNGY ADVVTEDPMSYNEEGWVVCRVFRKKNYQKIDDCPKITL SSLPDDTEEEKGPTFHNTQNVTGLDHVLLYMDRTGSNIC MPESQTTTQHQDDVLFMQLPSLETPKSESPVDQSFLTPS KLDFSPVQEKITERPVCSNWASLDRLVAWQLNNGHHNP CHRKSFDEEEENGDTMMQRWDLHWNNDDNVDLWSSF TESSSSLDPLLHLSV
FtNAC16	FtPinG0000381200.01	MNLSVNGQSQVPPGFRFHPTEEELLHYYLKKKVANQKI DLDVIQDIDLNKLEPWDIQEKCKIGSTPQNDWYFFSHKD KKYPTGTRTNRATTAGFWKATGRDKAICGSSRRIGMRK TLVFYKGRAPHGEKSDWIMHEYRLDDNIHELNVSNLSG DRDLGGDEGWVVCRVFRKKNYVKALHNPTNNRNSTH MMIRSKKHELDQDGFMHLPQLDSPSTNGVHEPIQQMNQ DGSDSMFNYEDDHNQEAPNSTVNWVELDRLVAYQLNG QASEASESTTNHLSCFNNHHLNHDSDITPFCLIPAGDYDR SNNNDRTTTATATTFQDLLRSQSSRVFDNDSIDDHFWSF TKQSSSPSSLDPISHFSV
GaNAC43	XP_0176296	MPENMSISVNGQSQVPPGFRFHPTEEELLQYYLRKKVSY

	05.1	EKIDLDVIRDVDLNKLEPWDIQESCKIGTTPQNDWYFFS HKDKKYPTGTRTNRATAAGFWKATGRDKVIYSNCRRIG MRKTLVFYKGRAPHGQKSDWIMHEYRLDDNIVETTVSN AMGEGTQEEGWVVCRIFKKKNHHKTLDNPNSSSLGSES RNHMLSACNEGELEQILEHMRRNCNEEGVANNSWRLM RPIETAISNSYPDSFMKLPSLESPNSTSSQNCYQPMIVDNE GSITNQMSGDPNSRLTNWAALDRLVASQLNGQTETSRQ LACFNDHSISMGYSNPSADHHHHDLQSPALRYNRSYNG TQDYNSEMDLWSLTRSSSSSDPLCHVVNASV
SmNAC43	QBZ39066.1	SQVPPGFRFHPTEEELLQYYLKKKVASEKIDLDVIQDVD LNKLEPWDIQEKCTIGSTPQNDWYFFSHKDKKYPTGTRT NRATASGFWKATGRDKVIYGNSRRIGMRKTLVFYKGRA PRGQKSDWIMHEYRLDDNTTANITHTNNHGSNAMGEG MQEEGWVVCRVFKKKNHSHKTLDTTPIISSSWSMLSTIN SCKEGTLEQMLQSIGTPFRRNHFPDSDNNTRFLTLSNLH DSPNSSSRTQNCYQSLHVQ
