# Supplementary material for: Tartary Buckwheat (Fagopyrum tataricum) NAC Transcription Factors FtNAC16 Negatively Regulates of Pod Cracking and Salinity Tolerant in Arabidopsis
Source: Int J Mol Sci. 2021 Mar 21;22(6):3197. doi: 10.3390/ijms22063197 (PMC8061773; doi:10.3390/ijms22063197)
Supplement: Supplementary file 1 [file ijms-22-03197-s001.zip › Supplementary Table S2. Data of evolution tree.pdf]

Supplementary Table S2. Data of evolution tree

Name	Group name	Gene ID	Sequence
FtNAC16	OSNAC7	FtPinG0000381200.01	MNLSVNGQSQVPPGFRFHPTEEELLHYYLKKKVANQKIDLDVIQDIDLNKLEPWDIQEKCKIGSTPQNDWYFFSHKDKKYPTGTRTNRATTAGFWKATGRDKAICGSSRRIGMRKTLVFYKGRAPHGEKSDWIMHEYRLDDNIHELNVSNLSGDRDLGGDEGWVVCRVFRKKNYVKALHNPTNNRNSTHMMIRSKKHELDQDGFMHLPQLDSPSTNGVHEPIQQMNQDGSDSMFNYEDDHNQEAPNSTVNWVELDRLVAYQLNGQASEASESTTNHLSCFNNHHLNHDSDITPFCLIPAGDYDRSNNNDRTTTATATTFQDLLRSQSSRVFDNDSIDDHFWSFTKQSSSPSSLDPISHFSV
AtNST1	OSNAC7	AT2G46770	MMSKSMSISVNGQSQVPPGFRFHPTEEELLQYYLRKKVNSIEIDLDVIRDVDLNKLEPWDIQEMCKIGTTPQNDWYFFSHKDKKYPTGTRTNRATAAGFWKATGRDKIIYSNGRRIGMRKTLVFYKGRAPHGQKSDWIMHEYRLDDNIISPEDVTVHEVVSIIGEASQDEGWVVCRIFKKKNLHKTLNSPVGGASLSGGGDTPKTTSSQIFNEDTLDQFLELMGRSCKEELNLDPFMKLPNLESPNSQAINNCHVSSPDTNHNIHVSNVVDTSFVTSWAALDRLVASQLNGPTSYSITAVNESHVGHDHLALPSVRSPYPSLNRSASYHAGLTQEYTPEMELWNTTTSSLSSSPGPFCHVSNGSG
AtNST2	OSNAC7	AT3G61910	MNISVNGQSQVPPGFRFHPTEEELLKYYLRKKISNIKIDLDVIPDIDLNKLEPWDIQEMCKIGTTPQNDWYFYSHKDKKYPTGTRTNRATTVGFWKATGRDKTIYTNGDRIGMRKTLVFYKGRAPHGQKSDWIMHEYRLDESVLISSCGDHDVNVETCDVIGSDEGWVVCRVFKKNNLCKNMISSSPASSVKTPSFNEETIEQLLEVMGQSCKGEIVLDPFLKLPNLECHNNTTITSYQWLIDDQVNNCHVSKVMDPSFITSWAALDRLVASQLNGPNSYSIPAVNETSQSPYHGLNRSGCNTGLTPDYYIPEIDLWNEADFARTTCHLLNGSG
AtNST3	OSNAC7	AT1G32770	MADNKVNLSINGQSKVPPGFRFHPTEEELLHYYLRKKVNSQKIDLDVIREVDLNKLEPWDIQEECRIGSTPQNDWYFFSHKDKKYPTGTRTNRATVAGFWKATGRDKIICSCVRRIGLRKTLVFYKGRAPHGQKSDWIMHEYRLDDTPMSNGYADVVTEDPMSYNEEGWVVCRVFRKKNY

			QKIDDCPKITLSSLPDDTEEEKGPTFHNTQN<br>VTGLDHVLLYMDRTGSNICMPESQTTTQHQ<br>DDVLFMQLPSLETPKSESPVDQSFLTPSKLDF<br>SPVQEKITERPVCSNWASLDRLVAWQLNNG<br>HHNPCHRKSFDEEEENGDTMMQRWDLHW<br>NNDDNVDLWSSFTESSSSLDPLLHLSV
AtVND1	OSNAC7	AT2G18060	MEPMESCSVPPGFRFHPTDEELVGYYLRKKI<br>ASQKIDLDVIRDIDLYRIEPWDLQEQCRIGYE<br>EQNEWYFFSHKDKKYPTGTRTNRATMAGF<br>WKATGRDKAVYDKTKLIGMRKTLVFYKGR<br>APNGKKSDWIMHEYRLESDENAPPQEEGW<br>VVCRAFKKRATGQAKNTETWSSSYFYDEVA<br>PNGVNSVMDPIDYISKQQHNIFGKGLMCKQ<br>ELEGMVDGINYIQSNQFIQLPQLQSPSLPLM<br>KRPSSSMSITSMDNNYNYKLPLADEESFESFI<br>RGEDRRKKKKQVMMTGNWRELDKFVASQL<br>MSQEDNGTSSFAGHHIVNEDKNNNDVEMDS<br>SMFLSEREEENRFVSEFLSTNSDYDIGICVFD<br>N
AtVND2	OSNAC7	AT4G36160.1	MESVDQSCSVPPGFRFHPTDEELVGYYLRK<br>KVASQKIDLDVIRDIDLYRIEPWDLQESCRIG<br>YEERNEWYFFSHKDKKYPTGTRTNRATMA<br>GFWKATGRDKAVYDKSKLIGMRKTLVFYK<br>GRAPNGQKTDWIMHEYRLESDENAPPQEEG<br>WVVCRAFKKKPMTGQAKNTETWSSSYFYD<br>ELPSGVRSVTEPLNYVSKQKQNVFAQDLMF<br>KQELEGSDIGLNFIHCDQFIQLPQLESPSLPLT<br>KRPVSLTSITSLEKNKNIYKRHLIEEDVSFNA<br>LISSGNKDKKKKKTSVMTTDWRALDKFVAS<br>QLMSQEDGVSGFGGHHEEDNNKIGHYNNE<br>ESNNKGSVETASSTLLSDREEENRFISGLLCS<br>NLDYDLYRDLHV
AtVND3	OSNAC7	AT5G66300	MMKVDQDYSCSIPPGFRFHPTDEELVGYYL<br>KKKIASQRIDLDVIREIDLYKIEPWDLQERCR<br>IGYEEQTEWYFFSHRDKKYPTGTRTNRATVA<br>GFWKATGRDKAVYLNSKLIGMRKTLVFYRG<br>RAPNGQKSDWIIHEYYSLESHQNSPPQEEG<br>WVVCRAFKKRTTIPTKRRQLWDPNCLFYDD<br>ATLLEPLDKRARHNPDFTATPFKQELLSEAS<br>HVQDGDFGSMYLQCIDDDQFSQLPQLESPSL<br>PSEITPHSTTFSENSSRKDDMSSEKRITDWRY<br>LDKFVASQFLMSGED
AtVND4	OSNAC7	AT1G12260	MNSFSHVPPGFRFHPTDEELVDYYLRKKVAS<br>KRIEIDFIKDIDLYKIEPWDLQELCKIGHEEQS

			DWYFFSHKDKKYPTGTRTNRATKAGFWKA<br>TGRDKAIYLRHSLIGMRKTLVFYKGRAPNG<br>QKSDWIMHEYRLETDENGTPQEEGWVVCR<br>VFKKRLAAVRRMGDYDSSPSHWYDDQLSF<br>MASELETNGQRRILPNHHQQQQHEHQQHM<br>PYGLNASAYALNNPNLQCKQELELHYNHLV<br>QRNHLLDESHLSFLQLPQLESPKIQQDNSNC<br>NSLPYGTSNIDNNSSHNANLQQSNIAHEEQL<br>NQGNQNFSSLYMNSGNEQVMDQVTDWRVL<br>DKFVASQLSNEEAATASASIQNNAKDTSNAE<br>YQVDEEKDPKRASDMGEEYTASTSSSCQIDL<br>WK
AtVND5	OSNAC7	AT1G62700	MNSFSQVPPGFRFHPTDEELVDYYLRKKVAS<br>KRIEIDIIKDVDLYKIEPCDLQELCKIGNEEQS<br>EWYFFSHKDKKYPTGTRTNRATKAGFWKAT<br>GRDKAIYIRHSLIGMRKTLVFYKGRAPNGQK<br>SDWIMHEYRLETSENGTPQEEGWVVCRVFK<br>KKLAATVRKMGDYHSSPSQHWYDDQLSFM<br>ASEIISSSPRQFLPNHHYNRHHHQQTLPCGL<br>NAFNNNNPNLQCKQELELHYNQMVQHQQQ<br>NHHLRESMFLQLPQLESPTSNCNSDNNNNT<br>RNISNLQKSSNISHEEQLQQGNQSFSSLYYD<br>QGVEQMTTDWRVLDKFVASQLSNDEEAAA<br>VVSSSSHQNNVKIDTRNTGYHVIDEGINLPE<br>NDSERVVEMGEEYSNAHAASTSSSCQIDL
AtVND6	OSNAC7	AT5G62380	MESLAHIPPGYRFHPTDEELVDYYLKNKVAF<br>PGMQVDVIKDVDLYKIEPWDIQELCGRGTG<br>EEREWYFFSHKDKKYPTGTRTNRATGSGFW<br>KATGRDKAIYSKQELVGMRKTLVFYKGRAP<br>NGQKSDWIMHEYRLETDENGPPHEEGWVV<br>CRAFKKKLTTMNYNNPRTMMGSSSGQESN<br>WFTQQMDVGNGNYYHLPDLESPRMFQGSS<br>SSSLSSLHQNDQDPYGVVLSTINATPTTIMQR<br>DDGHVITNDDDHMIMMNTSTGDHHQSGLL<br>VNDDHNDQVMDWQTLDKFVASQLIMSQEE<br>EEVNKDPSDNSSNETFHHLSEEQAATMVSM<br>NASSSSSPCSFYSWAQNTHT
AtVND7	OSNAC7	AT1G71930	MDNIMQSSMPPGFRFHPTEEELVGYYLDRKI<br>NSMKSALDVIVEIDLYKMEPWDIQARCKLG<br>YEEQNEWYFFSHKDRKYPTGTRTNRATAAG<br>FWKATGRDKAVLSKNSVIGMRKTLVYYKGR<br>APNGRKSDWIMHEYRLQNSELAPVQEEGW<br>VVCRAFRKPIPNQRPLGYEPWQNQLYHVES<br>SNNYSSSVTMNTSHHIGASSSSHNLNQMLM

			SNNHYNPNNTSSSMHQYGNIELPQLDSPSLSPSLGTNKDQNESFEQEEEKSFNCVDWRTLDTLLETQVIHPHNPNILMFETQSYNPAPSFPSMHQSYNEVEANIHHSLGCFPDS
AtSMB	OSNAC7	AT1G7958O	MEIGSSSTVAGGGQLSVPPGFRFHPTEEELLYYYLKKKVSYEPIDLDVIREVDLNKLEPWELKEKCRIGSGPQNEWYFFSHKDKKYPTGTRTNRATAAGFWKATGRDKSIHLNSSKKIGLRKTLVFYTGRAPHGQKTEWIMHEYRLDDSENEIQEDGWVVCRVFKKKNHFRGFHQEQEQDHHHHHQYISTNNDHDHHHHIDSNSNNHSPLILHPLDHHHHHHHIGRQIHMPLHEFANTLSHGSMHLPQLFSPDSAAAAAAAAASAQPFVSPINTTDIECSQNLLRLTSNNNYGGDWSFLDKLLTTGNMNQQQQQQVQNHQAKCFGDLSNNDNNDQADHLGNNNGGSSSSPVNQRFPFHYLGNDANLLKFPK
AtNAC070	OSNAC7	AT4G10350	MGSSSNGGVPPGFRFHPTDEELLHYYLKKKISYQKFEMEVIREVDLNKLEPWDLQERCKIGSTPQNEWYFFSHKDRKYPTGSRTNRATHAGFWKATGRDKCIRNSYKKIGMRKTLVFYKGRAPHGQKTDWIMHEYRLEDADDPQANPSEDGWVVCRVFMKKNLFKVVNEGSSSINSLDQHNHDASNNNHALQARSFMHRDSPYQLVRNHGAMTFELNKPDLALHQYPPIFHKPPSLGFDYSSGLARDSESAASEGLQYQQACEPGLDVGTCETVASHNHQQGLGEWAMMDRLVTCHMGNEDSSRGITYEDGNNNSSSVVQPVPATNQLTLRSEMDFWGYSK
NAC015	OSNAC7	AT1G33280	MSSSNGGVPPGFRFHPTDEELLHYYLKKKISYEKFEMEVIKEVDLNKIEPWDLQDRCKIGSTPQNEWYFFSHKDRKYPTGSRTNRATHSGFWKATGRDKCIRNSYKKIGMRKTLVFYKGRAPHGQKTDWIMHEYRIEDTEDDPCEDGWVVCRVFKKKNLFKVGNDVGSNISNNRLEARSFIRRESPYQGISMFELNKPEEISVHQYPQPPMFQPHHKPLSIGYDYSLALLPRESEYQQACQPSGVEVGTCKAVSEWGIVNCNMVSHEDSSRAMRFEDDGNNTSSTVQPPSNLLSLRGENGFLGLF
NAC1	NAC1	AT1G56010	METEEEMKESSISMVEAKLPPGFRFHPKDDELVCDYLMRRSLHNNHRPPLVLIQVDLNKCEPWDIPKMACVGGKDWYFYSQRDRKYATGLRTNRATATGYWKATGKDRTILRKGKLVGMRKTLVFYQGRAPRGRKTDWVMHEFRLQGSHH

			PPNHSLSSPKEDWVLCRVFHKNTEGVICRDN MGSCFDETASASLPPLMDPYINFDQEPSSYLS DDHHYIINEHVPCFSNLSQNQTLNSNLTNSV SELKIPCKNPNPLFTGGSASATLTGLDSFCSS DQMVLRALLSQLTKIDGSLGPKESQSYGEGS SESLLTDIGIPSTVWNC
NAC074	NAC1	AT4G28530	MGLKDIGSKLPPGFRFHPSDEELVCHYLCNK IRAKSDHGDVDDDDDDVDEALKGSTDLVEI DLHICEPWELPDVAKLNAKEWYFFSFRDRK YATGYRTNRATVSGYWKATGKDRTVMDPRT RQLVGMRKTLVFYRNRAPNGIKTTWIMHEF RLECPNIPPKEDWVLCRVFNKGRDSSLQDN NYYNNDNQTQRLEVNDAPDLNYNNQLPPL LSSPPHNHQHEKMKIQVCDQWEQLMKQPS RTTGHPYHHHCHHQTIACGWEQMMIGSLSS PSSHGPDHESLLNLLYVDNNNSVNISGDHHQ NYEKILLSSLDMTSLDHDKTCMGSSSDGGM VSDLHMECGGLSFETENILAFQ
NAC096	ANAC011	AT5G46590	MGSSCLPPGFRFHPTDEELIEYYLKRKVEGL EIELEVIPVIDLYSFDPWELPDKSFLPNRDME WYFFCSRDKKYPNGFRTNRGTKAGYWKAT GKDRKITSRSSSIIGYRKTLVFYKGRAPLGDR SNWIMHEYRLCDDDTSQGSQNLKGAFVLCR VAMKNEIKTNTKIRKIPSEQTIGSGESSGLSSR VTSPSRDETMPFHSFANPVSTETDSSNIWISP EFILDSSKDYPQIQDVASQCFQQDFDFPIIGN QNMEFPASTSLDQNMDEFMQNGYWTNYGY DQTGLFGYSDFS
NAC071	ANAC011	AT4G17980	MGSSCLPPGFRFHPTDEELIGYYLSRKIEGLE IELEVIPVIDLYKFDPWELPGKSFLPNRDLEW FFFCPRDKKYANGSRTNRATKAGYWKATGK DRKITCKSSHVIAGYRKTLVFYEGRAPLGDR TNWFMHEYRLCDIDDHSQKSPNFKGAFALC RVVKKNELKKNSKSLKNKNEQDIGSCYSSL ATSPCRDEASQIQSFKPSSTTNDSSSIWISPDFI LDSSKDYPQIKEVASECFPNYHFPVTTANHH VEFPLQEMLVRS
NAC011	ANAC011	AT1G32510	MVGSFLPPGFRFYPTDEELVGYYLHRRNEGL EIELEIIPLMDLYKFDPWELPEKSFLPNRDME WFFFCHRDRKYQNGSRINRATKSGYWKATG KDRKIVCHSSSSSSSSSITGCRKTLVFYMGRA PFGGRTEWVMHEYRLFDNDTSQGSLNFKGD FALCRVIKRNEHTLKKCEIISPEVSDESLSNN VNNFCQASDLEKGSCDASNTRLSSPDFILESS

			FQGNSHSKTEEDSGFQVFTLPEFEYPLEVFADLNFDLEMEDPFMFDYHPEPHMNNEVMSHHIRG
NAC020	ANAC011	AT1G54330	MAPMSLPPGFRFHPTDEELVAYYLDRKVNGQAIELEIIPEVDLYKCEPWDLPEKSFLPGNDMEWYFYSTRDKKYPNGSRTNRATRAGYWKATGKDRTVESKKMKMGMKKTLVYYRGRAPHGLRTNWVMHEYRLTHAPSSSLKESYALCRVFKKNIQIPKRKGEEEEAEEESTSVGKEEEEEKEKKWRKCDGNYIEDESLKRASAETSSSELTQGVLLDEANSSSIFALHFSSSLLDDHDHLFSNYSHQLPYHPPLQLQDFPQLSMNEAEIMSIQQDFQCRDSMNGTLDEIFSSSATFPASL
