# Supplementary material for: Tartary Buckwheat (Fagopyrum tataricum) NAC Transcription Factors FtNAC16 Negatively Regulates of Pod Cracking and Salinity Tolerant in Arabidopsis
Source: Int J Mol Sci. 2021 Mar 21;22(6):3197. doi: 10.3390/ijms22063197 (PMC8061773; doi:10.3390/ijms22063197)
Supplement: Supplementary file 1 [file ijms-22-03197-s001.zip › Supplementary Table S3. q-PCR Primers.pdf]

Supplementary Table S3. q-PCR Primers used in this study

Gene Name	Gene No.		Sequences (5´ to 3´)
PAL1	At2g37040	Forward	CAGGATCAAGGAATGTAGGTCG
		Reverse	AAATCGCCGTGAAAACCTTG
PAL2	At3g53260	Forward	CGTAGACCAGTCGTGAATCTTG
		Reverse	TCTCCATAACCCAATCACTGC
PAL3	At5g04230	Forward	AGCGGTTAATGAGGTTGTGAG
		Reverse	TTAAAGGGTTAGTGAGGCTGC
C4H	At2g30490	Forward	CGGATCTAACAAAGGAAGTGCTC
		Reverse	CCAATGCTCGCCGTAAACAG
CCoAOMT1	At4g34050	Forward	CTGGCTATGGATGTCAACAGAG
		Reverse	ATCAAGAACGGGAAGAGCAG
4CL1	At1g51680	Forward	TGCCTAATCAACGGACCAAC
		Reverse	AATTCGGGACAGTTTGGGAG
4CL2	At3g21240	Forward	CTATGGGATGACAGAAGCAGG
		Reverse	GTATCTTCATCTCGGCGTTCC
CAD5	At4g34230	Forward	AATCTGCCACACCGATCTTC
		Reverse	AACCAACTCCAACTATGTCCC
CAD6	At4g37970	Forward	CAAATGAAGGCTGCAATGGG
		Reverse	ACCGAGTAGAACAAGTTTACCG
CCR1	At2g02400	Forward	TAGGCTTAGAGTTCACTTCCACCA
		Reverse	CCACGGATTCTTGCGATGC
CCR2	At2g33600	Forward	CGGCGGCTACATTGCTTCT
		Reverse	TGAGTCTTTCCTTGGCTCCTTG
PRX52	At5g05340	Forward	CTTCTACTCAACCTCTTGCCC
		Reverse	CCGTTGACAAAGCAATCGTG
F5H	At4g36220	Forward	GGAGGAACGGAAACGGTAGC
		Reverse	CTTCGGCGAGTTCTTGTTGG

COMT1	At5g54160	Forward	CAAGCCTCTCAACCAAACAAG
		Reverse	TGCCTCAAACTCTTTCTCGG
sAPX	At4g08390	Forward	TCCTCCTTCACCTGCTACTCATC
		Reverse	CCAACCACTACGTTCTGGCCTA
Actin2	At3g18780	Forward	GTATGAGCAAAGAAATCACAGCACT
		Reverse	CTGAGGGAAGCAAGAATGGAAC
ABI2	At5g57050	Forward	TGACGGAGGAGATAGTGAAGGAG
		Reverse	CCACCGAGGTAGACCCAACAGT
HKT1;1	At4g10310	Forward	TCAACGTCCTTAACATCACTCTCG
		Reverse	GACTCCATCGTCCTGCAAACC
SOS1	At2g01980	Forward	CGGCAGCATGGTTAATGTGTAC
		Reverse	TTGGCTGAAACGAGACCTTGA

Gene Name	Gene No.		Sequences (5´ to 3´)
FtNAC16	FtPinG0000381200.01	Forward	AGCTTCAACCGCGAACTGG
		Reverse	GACTCACCGTATGCTCCTCTGG
FtActin	FtPinG0002124000.01	Forward	GGAAGTATAGCGTCTGGATTGGC
		Reverse	CACTTGCGGTGAACGATTGC
