# Supplementary material for: Tartary Buckwheat (Fagopyrum tataricum) NAC Transcription Factors FtNAC16 Negatively Regulates of Pod Cracking and Salinity Tolerant in Arabidopsis
Source: Int J Mol Sci. 2021 Mar 21;22(6):3197. doi: 10.3390/ijms22063197 (PMC8061773; doi:10.3390/ijms22063197)
Supplement: Supplementary file 1 [file ijms-22-03197-s001.zip › Supporting Information.pdf]

Supporting Information:

Fig. S1. Phloroglucinol-HCl Stain of *A. thaliana* fruit pods. *A. thaliana* fruit pods were cross-cut, lignin was stained with phloroglucinol, and the cracking area of the black box was locally enlarged.

Fig. S2. Relative expression of the *FtNAC16* transcript in Tartary buckwheat subjected to ABA. The relative expression of the *FtNAC16* transcript in Tartary buckwheat was determined by qRT-PCR. Two-week-old seedlings were used to extract mRNA following treatment with 150 μM ABA for 9 h. The sampling time was 0, 0.5, 1, 3, 6, and 9 h. The error bars indicate the standard error (SE) of three replicates. ** $P < 0.01$.

Fig. S3. Expression of key enzyme genes in the lignin pathway after salt stress treatment in WT and OE-FtNAC16.

Fig. S4. Expression of the *AtABI4* gene in the lignin pathway after salt stress treatment in WT, *nst1/3* and *nst1/3*-FtNAC16. ** $P < 0.01$.

Table. S1. Alignment sequence

Table. S2. Data of evolution tree

Table. S3. q-PCR Primers
