# Supplementary figures and images for: Tartary Buckwheat (Fagopyrum tataricum) NAC Transcription Factors FtNAC16 Negatively Regulates of Pod Cracking and Salinity Tolerant in Arabidopsis
Source: Int J Mol Sci. 2021 Mar 21;22(6):3197. doi: 10.3390/ijms22063197 (PMC8061773; doi:10.3390/ijms22063197)

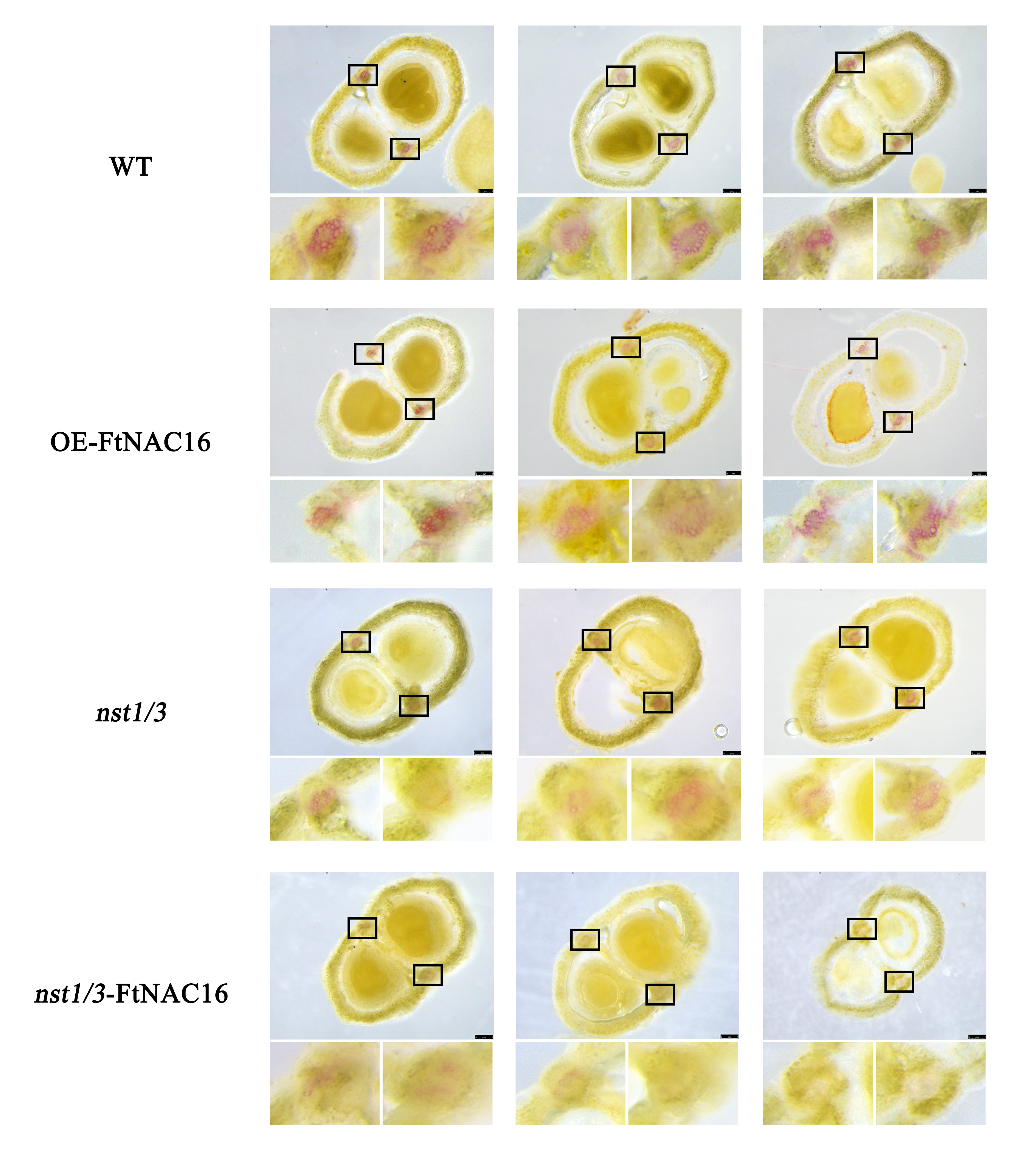

Supplement: Supplementary file 1 [file ijms-22-03197-s001.zip › Supplementary Figure.1.tif]

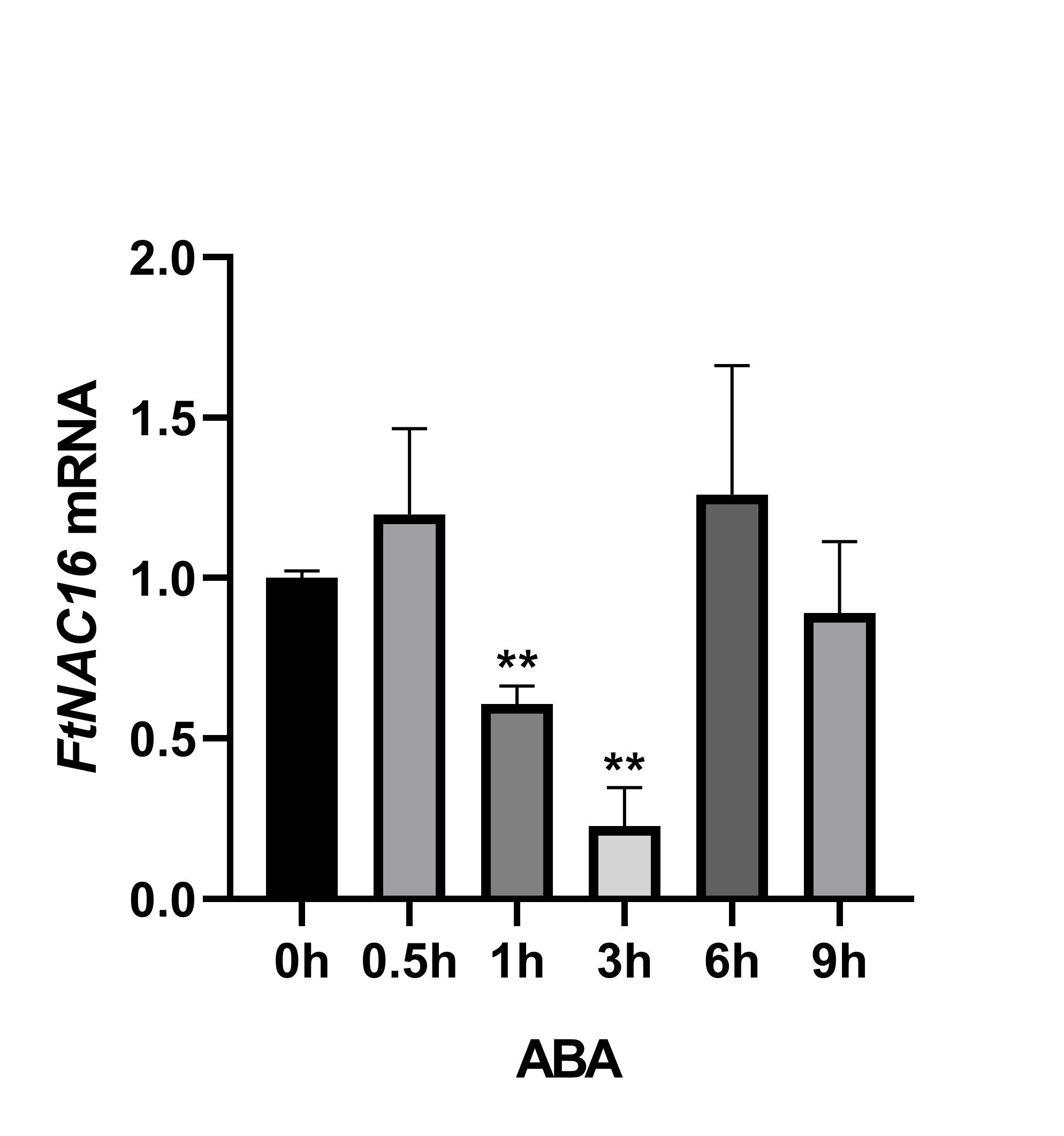

Supplement: Supplementary file 1 [file ijms-22-03197-s001.zip › Supplementary Figure.2.tif]

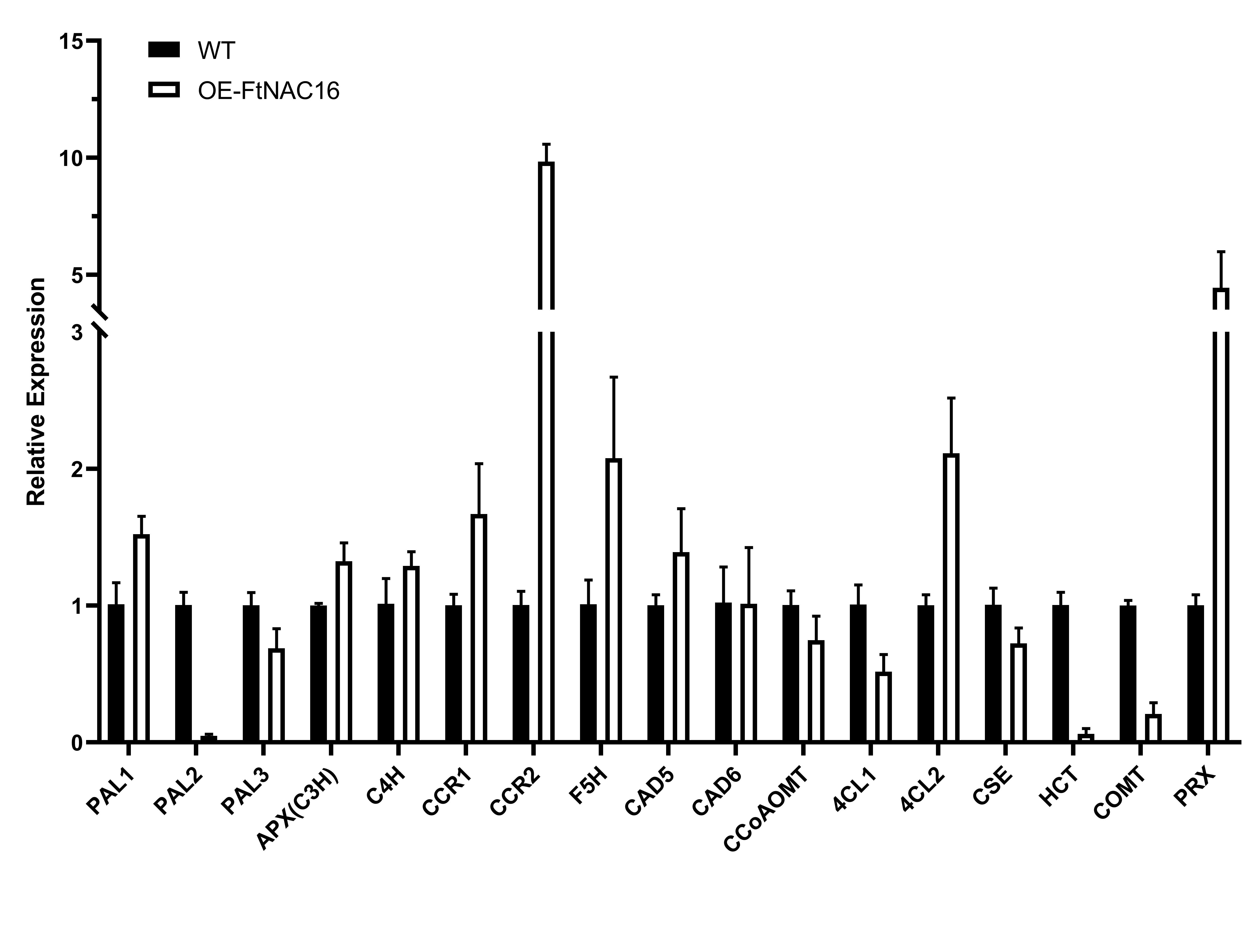

Supplement: Supplementary file 1 [file ijms-22-03197-s001.zip › Supplementary Figure.3.tif]

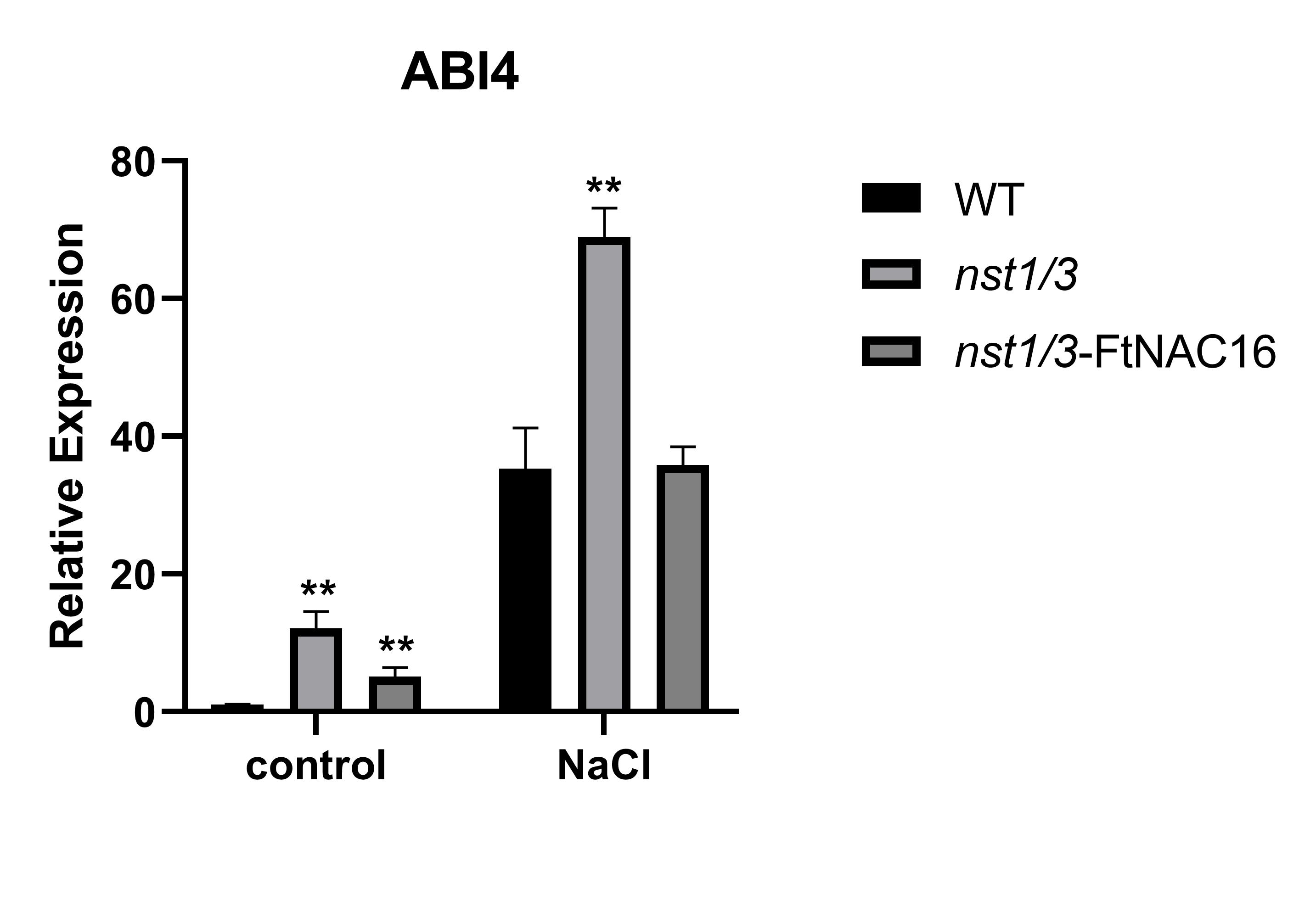

Supplement: Supplementary file 1 [file ijms-22-03197-s001.zip › Supplementary Figure.4.tif]
